# The use of dupilumab in severe atopic dermatitis during pregnancy: a case report

**DOI:** 10.1186/s13223-022-00650-w

**Published:** 2022-02-03

**Authors:** Nabeel H. Akhtar, Touraj Khosravi-Hafshejani, Daud Akhtar, Gurbir Dhadwal, Amin Kanani

**Affiliations:** 1 School of Medicine, University College Dublin, Dublin, Ireland; 2 Department of Dermatology and Skin Science, University of British Columbia, Vancouver, Canada; 3 Department of Medicine, University of British Columbia, Vancouver, Canada; 4 Department of Medicine, Division of Allergy and Immunology, University of British Columbia, Vancouver, Canada

**Keywords:** Atopic dermatitis, Dupilumab, Pregnancy, Outcome

## Abstract

**Background:**

Atopic dermatitis is a common chronic skin disease that can occur in pregnancy. Current treatments include topical and systemic glucocorticoids and cyclosporine. Presently, the only biologic approved for atopic dermatitis is dupilumab with limited data available regarding its safety profile in pregnancy.

**Case presentation:**

We report a case of severe atopic dermatitis treated safely with dupilumab with no adverse maternal or fetal outcomes and resolution of atopic dermatitis postpartum in the absence of maintenance dupilumab therapy.

**Conclusion:**

Here we demonstrate the safe use of dupilumab in pregnancy. Further research is needed to elucidate the role of dupilumab in the management of atopic dermatitis during pregnancy.

## Introduction

Atopic dermatitis (AD), a chronic inflammatory skin disorder that manifests with severe pruritis in the setting of eczematous lesions, is one of the most common skin disorders in adults and children. Globally, the prevalence of AD continues to increase with up to 20% of children and 3% of adults affected [1]. In pregnancy, AD is the most common skin disease accounting for 36–59% of all dermatoses [2, 3]. AD poses a significant economic burden with an estimated annual cost in Canada of $1.4 billion dollars [4]. Additionally, AD results in significant morbidity and adversely affects quality of life [5–7]. Current treatment options for moderate to severe AD in pregnancy include oral corticosteroids, azathioprine, cyclosporine and phototherapy [3, 8]. Presently, the only biologic approved for moderate to severe AD is dupilumab. Dupilumab is a fully human monoclonal antibody of the immunoglobulin G(IgG)4 subclass that exerts its effect through the disruption of interleukin (IL)-4 and IL-13 signaling pathways, key modulators in the pathogenesis of AD [9]. Specifically it binds to the IL-4 receptor alpha subunit, which is shared by the IL04 and IL-13 receptor complexes which leads to the subsequent inhibition of IL-4 signaling via the type 1 receptor and both IL-4 and IL-13 via the type 2 receptor. Placental transfer of maternal IgG antibodies to the fetus is an important mechanism that provides protection to an infant. Dupilumab, like other IgG antibodies, is expected to cross the placental barrier with the highest chance of transfer after 2 trimesters. There remains a paucity of data evaluating the impact of dupilumab in the pregnant population and the subsequent effects on fetal outcomes [10]. We describe a case of severe AD treated safely with dupilumab during pregnancy with subsequent resolution of symptoms in the post-partum period.

## Case report

A 33-year-old gravida 1 para 1 with a past medical history of seasonal rhinitis and severe AD dating back to early childhood, presented in the setting of worsening AD refractory to high potency topical corticosteroids, systemic corticosteroids, antibiotics for staphylococcal superinfection, methotrexate, cyclosporine as well as ultraviolet phototherapy. Her initial diagnosis and care were directed by a different healthcare provider 4 years prior to examination at our institution. Her examination at that time revealed periorbital hyperpigmentation, erythematous lichenification of the anterior chest, arms, and neck. The back was spared. She was subsequently started on hydroxyzine, clarithromycin and triamcinolone cream which resulted in mild improvement, but on discontinuation her AD flared. Three years after multiple therapies she sought treatment in the United States. Her Investigator Global Assessment for Atopic Dermatitis score of 4 was indicative of severe disease with greater than 10% total body surface area involvement. She was subsequently initiated on dupilumab 300 mg every 2 weeks with significant improvement in her skin disease and quality of life. She became pregnant 12 months later. Discussions about continuing dupilumab during pregnancy were held with both her allergist and dermatologist reviewing the risks and benefits of therapy during pregnancy as well as concerns regarding maternal antibodies crossing the placenta. She decided to remain on dupilumab 300 mg every 2 weeks. During her pregnancy, the patient had routine monitoring including regular bloodwork and guideline-based ultrasounds which were normal during the first 2 trimesters. The patient continued use of lubricating eye drops and thus, did not develop ocular symptoms during her pregnancy. At 27 weeks gestation, the patient self-discontinued dupilumab. Unfortunately, she experienced a self-reported severe flare of AD only 2 weeks after discontinuation that was refractory to topical treatments and the patient self reinitiated dupilumab at 29 weeks gestation. The patient was not assessed in clinic at this time and therefore there are no severity scores available for this flare. The patient remained on dupilumab until 36 weeks of gestation. At 38 weeks gestational age, ultrasound imaging revealed concerns for intrauterine growth restriction as well as breech position. The patient underwent urgent Caesarean Section with delivery of a healthy female infant (Weight 2480 g). Surgical pathology revealed normal umbilical cord vasculature, normal villous maturation compatible with a third trimester placenta with no evidence of villitis or infarct, and no chorioamnionitis. There were no issues postpartum. The patient was reassessed 6 weeks postpartum and had opted to breastfeed. The patient did not develop any further flares of AD despite self discontinuation of dupilumab. To date post-partum, the infant continues to meet age appropriate milestones.

## Discussion and conclusion

Immunity in pregnancy is characterized by a shift from cell-mediated immunity (Th1) to humoral immunity (Th2) and is associated with a reduction in fetal demise/abortion. The upregulation of the Th2 response is similarly seen in atopic diseases which are associated with reduced fertility. It is, therefore, not surprising that pregnancy worsens AD severity. For this reason, adequate control of AD in pregnancy is warranted and may reduce the risk of severe complications such as eczema herpeticum, bacterial infections, and improve quality of life with reduced psychosocial comorbidities [11–13].

The current cornerstones of AD treatment in pregnancy include topical corticosteroids, topical calcineurin inhibitors, and narrowband ultraviolet B (UVB). In the setting of refractory disease, cyclosporine or glucocorticoids can be used as alternative therapies for long term disease control, although the risk of low birth weight in neonates with maternal cyclosporine or long term steroid use must be considered [3, 9].

Biologic therapies in pregnancy continues to gain traction especially in the management of inflammatory bowel disease, and rheumatoid arthritis where the use of tumor necrosis factor (TNFα) inhibitors is generally considered safe in the first 2 trimesters due to the negligible transfer of maternal antibodies during this period [10]. With regards to AD, there remains a paucity of data with only 2 case reports reporting the use of dupilumab in pregnancy. Both case reports demonstrated favorable maternal and fetal outcomes [11, 14]. The effects of dupilumab on breastfeeding infants is unclear and, therefore, is not currently recommended in lactating women [8]. Similarly, the effects of dupilumab on the neonatal immune system are uncertain and the potential for altered newborn immunity exists [10]. For this reason, the administration of live, attenuated vaccines should be delayed for at least 6 months post delivery [10]. In this case, the patient demonstrated well controlled AD despite discontinuing dupilumab post-partum.

Shared decision making is an important process in patient care especially for the pregnant population as studies of therapeutic safety and efficacy are limited in this population group. A discussion regarding the course of AD and therapeutic options during pregnancy should ideally occur pre-partum. Shared decision making should continue during pregnancy and postpartum and should involve continued education of patients on their treatment options, discussing the benefits and risks to maternal and fetal health, and understanding the patients’ values and preferences [15].

This case report adds to current literature regarding the use dupilumab in pregnancy. To our knowledge, this is the first case report of a pregnant patient with AD treated with dupilumab in Canada. Our case is the first to demonstrate symptom resolution without re-initiation of dupilumab in the postpartum setting with no adverse fetal or maternal events.

## Data Availability

Patient health records have been stored in a secure facilty and are available on request.

## Abbreviations

AD: Atopic dermatitis
Igg: Immunoglobulin
IL: Interleukin
Th1: Cell mediated immunity
Th2: Humoral immunity
UVB: Narrowband ultraviolet B
